# Human cytokine-induced killer cells have enhanced in vitro cytolytic activity via non-viral interleukin-2 gene transfer

**DOI:** 10.1186/1479-0556-2-12

**Published:** 2004-08-25

**Authors:** Srinivas Nagaraj, Carsten Ziske, Ingo GH Schmidt-Wolf

**Affiliations:** 1 Department of Internal Medicine I, General Internal Medicine Rheinische Friedrich-Wilhelms-Universität, Bonn, Germany

**Keywords:** IL-2, gene therapy, dendritic cells, CIK

## Abstract

Modulation of the immune system by genetically modified immunological effector cells is of potential therapeutic value in the treatment of malignancies. Interleukin-2 (IL-2) is a crucial cytokine which induces potent antitumor response. Cytokine-induced killer cells (CIK) have been described as highly efficient cytotoxic effector cells capable of lysing tumor cell targets and are capable of recognizing these cells in a non-MHC restricted fashion. Dendritic cells (DC) are the major antigen presenting cells. This study evaluated the antitumor effect of CIK cells which were non-virally transfected with IL-2 and co-cultured with pulsed and unpulsed DC. Human CIK cells generated from peripheral blood were transfected *in vitro *with plasmid encoding for the human IL-2. Transfection involved a combination of electrical parameters and a specific solution to deliver plasmid directly to the cell nucleus by using the Nucleofector^® ^electroporation system. Nucleofection resulted in the production of IL-2 with a mean of 478.5 pg/10^6 ^cells (range of 107.6–1079.3 pg /10^6 ^cells/24 h) compared to mock transfected CIK cells (31 pg/10^6 ^cells) (*P *= 0.05). After co-culturing with DC their functional ability was assessed *in vitro *by a cytotoxicity assay. On comparison with non-transfected CIK cells co-cultured with DCs (36.5 ± 5.3 %), transfected CIK cells co-cultured with DC had a significantly higher lytic activity of 58.5 ± 3.2% (*P *= 0.03) against Dan G cells, a human pancreatic carcinoma cell line.

## Introduction

Advances in the characterization of cytokines and tumor antigens, coupled with our increasing ability to manipulate gene expression, have fostered a new era of tumor immunotherapy [1]. Interleukin-2 (IL-2) affects a variety of components of the cellular immune system, including B cells and macrophages by inducing the secretion of tumor necrosis factors (TNF) α and β and interferon-γ. Mainly, IL-2 is responsible for the proliferation of T cells. In animal and some human studies, systemic administration of IL-2 has antitumor effects, mediated by cytotoxic effector cells (such as lymphokine-activated killer – LAK cells and cytotoxic T lymphocytes) [2]. Such systemic administration often induces high toxicity and is shown to be inferior to local continuous production of cytokine, for recruitment of T cells [3]. Cytokine-induced killer cells (CIK) are non-major histocompatibility complex-restricted cytotoxic lymphocytes generated by incubation of peripheral blood lymphocytes with anti-CD3 monoclonal antibody, interleukin (IL)-2, IL-1 and interferon gamma (IFN-γ). CIK represent cells with high antitumor cytotoxicity in vitro and in vivo [4]. CIK cells possess enhanced cytotoxic activity as compared to standard lymphokine activated killer (LAK) cells [5,6]. CIK cells, express CD4 (45.4+/-3.2) % and CD8(47.7+/-11.0%) markers. It has been shown that NKT cells co-expressing CD3 and CD56 markers on their surface represent the major cytotoxic subset of CIK cells [7]. These NKT cells are derived from T cells [5]. Because of the increase in cytotoxicity and high proliferative response, CIK cells have a 73-fold increase in total lytic units per culture as compared to IL-2-stimulated LAK cells. Gene transfers of cytokine genes to CIK and tumor cells have been extensively studied. CIK cells transfected with cytokine genes have shown to induce antitumor effects [8].

Dendritic cells (DC) are specialized antigen-presenting cells located throughout the human body. They represent heterogeneous cell population, residing in most peripheral tissues where they represent 1–2% of the cell numbers. In the absence of ongoing inflammatory and immune responses, dendritic cells constitutively patrol through the blood, peripheral tissues, lymph and secondary lymphoid organs [9]. Morphologically, mature DC are large cells with elongated and stellated processes. They express high levels of MHC I and II, CD11 a, b, c, CD40, CD54, CD58, CD80, CD83, CD86. The most typical markers at present are MHC I, II and co-stimulatory markers such as CD80, CD86, [10] which present signals to CD4 and CD8 positive T cells. Once T cells are activated after interaction with a DC exhibiting the appropriate tumor-associated peptide antigen and class I molecule, they kill other cells that express these molecules such as tumor cells.

Exocrine pancreatic carcinomas have a very poor prognosis and a resistance to conventional therapy. This is mainly induced by lack of immuno-competent cells. Therefore, it might be beneficial for patients with pancreatic cancer to induce an immune attack against the tumor by inserting a cytokine gene into the immunological effector cells. The aim of this study was to evaluate the antitumor immune responses of a cytokine immunotherapy using gene transfer to provide continuous and local cytokine production and therefore showing an improved cytotoxic effect against pancreatic cancer cells.

## Material and methods

### Generation of dendritic cells

DC were generated as described before [4,7]. Blood was drawn according to our protocol accepted by the local ethics committee from healthy volunteers. Briefly, peripheral blood lymphocytes were isolated from buffy coats by Ficoll density gradient centrifugation (Lymphoprep, Nycomed, Oslo Norway). These cells were allowed to adhere in six-well-plates at a density of 5 × 10^6 ^cells/ml for one hour at 37°C in complete RPMI 1640 with 10% heat-inactivated fetal calf serum, 100 U/ml penicillin and 100 μg/ml streptomycin. The non-adherent cells were collected for generating CIK cells. The adherent cells were cultured in 2 ml RPMI 1640 with autologous, heat-inactivated serum, 750 IU GM-CSF and 500 IU IL-4 (Essex Pharma, Nürnberg, Germany), 100 U/ml penicillin and 100 μg/ml streptomycin per well for seven days for generating DC. The media along with the necessary cytokines were changed every third day.

### Generation of CIK

CIK cells were generated as described previously [11]. In brief, non-adherent Ficoll separated human peripheral blood mononuclear cells derived from healthy individuals were prepared and grown in RPMI 1640 medium (Gibco BRL, Berlin, Germany), containing 10% fetal calf serum (Gibco BRL), 25 mM Hepes, 100 U/ml penicillin and 100 μg/ml streptomycin. One thousand IU/ml human recombinant interferon γ (Boehringer Mannheim, Germany) was added on day 0. After 24 hrs of incubation, 50 ng/ml of an anti-CD3 (Orthoclone OKT 3, Cilag GmbH, Sulzbach, Germany), 100 U/ml interleukin-1β and 300 U/ml interleukin-2 (R and D Systems, Wiesbaden, Germany) were added. Cells were incubated at 37°C in a humidified atmosphere of 5% CO_2 _and sub-cultured every third day in fresh complete medium with 300 U/ml IL-2 at 3 × 10^6 ^cells/ml. CIK cells were harvested on day +7 and were co-cultured for seven days with autologous DC at a stimulator (DC) to responder (CIK) ratio of 1:5.

### Cell lines

The human pancreatic carcinoma cell line DAN-G was purchased from DSMZ (Deutsche Sammlung für Zellkultur, Braunschweig, Germany). The cells were maintained in RPMI 1640 supplemented with 10% fetal calf serum (FCS, PAA) 100 U/ml penicillin and 100 μg/ml streptomycin (Seromed, Jülich, Germany) and grown at 37°C in a humidified atmosphere of 5% CO_2_.

### Preparation of IL-2 plasmid

cDNA of human IL-2 was cloned in the plasmid pMTV.05 (Invitrogen, Karlsruhe, Germany). The recombinant pMTv-hIL-2 (referred to as pIL-2) was transformed and the plasmid was eluted using a mini-prep column (Qiagen GmbH, Hilden Germany) according to the manufacturer's protocol.

### Pulsing of DC

DCs were pulsed with tumor lysate of Dan-G cells on day +5 [12].

### Gene transfer by nucleofection

CIK cells were subjected to a combination of electrical parameters and specific solution to deliver the DNA directly to the cell nucleus under mild conditions by using a commercially available nucleofection system on day 10 according to manufacturer's protocol (Nucleofector Amaxa Biosystems GmbH, Cologne, Germany). Five times 10^6 ^CIK cells were nucleofected in an electroporation cuvette along with pre-warmed nucleofector solution and 3 μg of pMTV-hIL-2 using the programme U-14. Once nucleofected, CIK cells were transferred into fresh pre-warmed media with the necessary cytokines and serum.

### IL-2 measurement

Cell culture supernatants from the nucleofected and non-nucleofected CIK cells were sampled at 24 hrs and 48 hrs, respectively. An enzyme linked immunosorbent assay for IL-2 with matched antibody pairs was performed according to the manufacturer's instructions (R and D Systems, Wiesbaden, Germany).

### Cytotoxicity assay

A DELFIA EuTDA^® ^non-radioactive cytotoxicity assay was used as a fluorometric alternative to the ^51^Cr release assay (Perkin Elmer Wallac Life Sciences, Brussels, Belgium). The assay is based on loading target cells with a fluorescence enhancing ligand. After cytolysis the ligand is released and introduced to the DELFIA^® ^Europium solution. The measured signal correlates directly with the amount of lysed cells [13]. Each experiment was performed in triplicates and the mean value was calculated. After incubation, 20-microl aliquots from all wells are transferred to a fresh 96-well plate. To each well of the plate, 180 microl of the Europium solution mix is added and incubated at room temperature for 15 min on a shaker. Fluorescence data are collected using a 96-well plate in a time-resolved fluorometer (PerkinElmer, Brussels, Belgium). Maximum release was obtained by incubating Dan-G cells with 1% lysis Buffer (Perkin Elmer Wallac Life Sciences, Brussels, Belgium). Target cells without effector cells are used as negative control (spontaneous release). Specific releases are calculated as percentage cytotoxicity = experimental release (counts) minus spontaneous release (counts) divided through maximum release (counts) minus spontaneous release (counts) of target cells.

Students't' test was applied. A *P *value < 0.05 was considered significant.

## Results

### In vitro generation of DC and CIK

DC were generated from CD14^+ ^monocytes using GM-CSF and IL-4. Adherent cells showed cytoplasmic processes typical for DC. After co-culture with CIK cells they formed typical cluster. Flow cytometry showed CD14 negative populations, expressing markers typical for DC (CD80^+^, CD83^+^, CD86^+ ^and HLA-DR expressing cells). The CIK cells were phenotyped with antibodies against CD3, CD8, CD16, CD40L, CD56, HLA-ABC and HLA-DR. Data was similar to other studies by our group (data not shown) [5,11,14].

### Transfection efficiency

**Figure 1:**
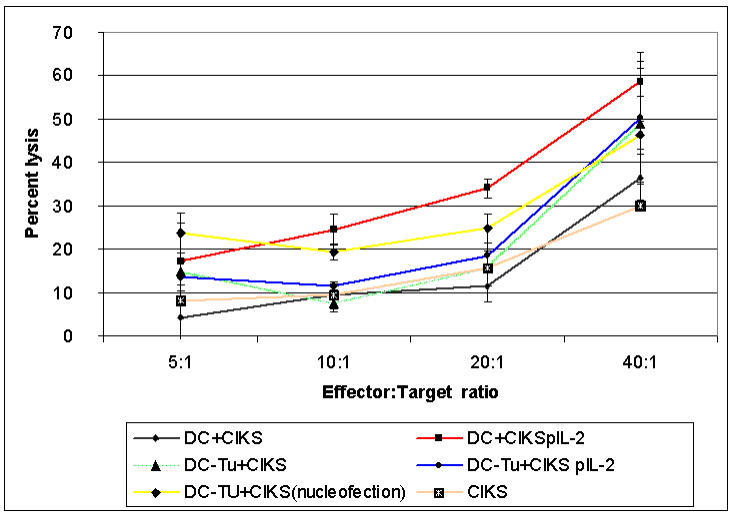
Cytotoxic activity of immunological effector cells that had been co-cultured with DC against Dan-G pancreatic carcinoma cells. Immunological effector cells from a donor were co-cultured from days +10 to +14 with autologous DC cultures seven days of age, as described in materials and methods. DC were pulsed at day +7 with 200 ng/ml of tumor lysate. Cytotoxic activity at various effector to target cell ratios was measured by Europium release assay. Dan-G cells were used as targets. Data represent results of three separate experiments and are shown as mean. CIKs = CIKS cells only DC+CIKS = naive DC co-cultured with CIK cells DC+CIKSpIL-2 = naive DC co-cultured with CIK cells nucleofected with pIL-2 DC-Tu+CIKS = DC pulsed with tumor lysate and co-cultured with CIK cells DC-Tu+CIKSpIL-2 = DC pulsed with tumor lysate and co-cultured with CIK cells nucleofected with pIL-2 DC-Tu+CIKS (nucleofected) = DC pulsed with tumor lysate and co-cultured with CIK cells nucleofected without plasmid

Transfection efficiency was determined by eGFP expression analysis using a fluorescence activated cell sorter. Viable cells were determined by propidium iodide staining. Nucleofection efficiency for eGFP gene transfer into the stimulated CIK cells resulted in a transient expression of 43 +/- 3.8% of the cells after 24 hours. 17% of the cells were not viable after transfection. The amount of IL-2 was the maximum after 24 hrs. An irrelevant plasmid containing eGFP was nucleofected and compared to the plasmid containing the IL-2 insert in various samples (n = 8). Nucleofection resulted in the production of IL-2 with a mean of 478.5 pg /10^6 ^cells (range of 107.6–1079.3 pg /10^6 ^cells/24 h) compared to irrelevantly transfected (containing eGFP) CIK cells (31 pg/10^6 ^cells) (*P *= 0.05). CIK cells secreting IL-2 were co-cultured from days +7 to +14 with DC, 10 days of age. Cytotoxicity of effector cells was analyzed. Co-culture of effector cells with DC led to an increase in cytotoxic activity as measured in a Eu-release assay using Dan-G. Eu-release in co-cultured CIK cells transfected with pIL-2 and DC was 58.5 ± 3.2% at an effector:target ratio of 1:40 (Fig. 1) compared to non-transfected CIK cells co-cultured with DC (36.5 ± 5.3%, *P *= 0.03). In order to further enhance cytotoxic activity DC were pulsed with tumor lysate of Dan-G cells on day +5. However, lytic activity (50.3%) was not significantly enhanced (*P *= 0.33) when compared to non-transfected cells (Fig. 1). Lytic activity of DCs pulsed with tumor lysate and co-cultured with non-transfected CIK cells was 48.9% where as DC pulsed with tumor lysate and co-cultured with CIK cells transfected without the plasmid was 46.3% and CIK cells alone 40.3% (Fig. 1).

## Discussion

In this report, transfection of CIK cells with IL-2 demonstrated a prominent augmentation of antitumor immunity *in vitro *against pancreatic carcinoma cell lines via secreting significant amounts of IL-2.

Ductal pancreatic adenocarcinoma is the fourth leading cause of cancer death in the Western world. Unfortunately, recent advances in diagnostics, staging, and therapy have not resulted in significant improvements. Thus, new approaches are necessary to improve the outcome of patients with exocrine pancreatic cancer. CIK cells are the most potent mediators of the lyses of autologous and allogeneic cancer cells *in vitro *in a non MHC restricted fashion [15], have a higher antitumor toxicity as compared to standard lymphokine activated cells [5,6,15] and may be suitable to remove tumor cells resistant to chemotherapy [8]. Therefore, they are ideal candidates for further enhancing cytotoxic activity. CIK cells have been shown to upregulate DC specific markers [16]. Transgene candidates to potentially activate systemic immune response include genes encoding for co-stimulatory molecules, lymphotactic chemokines, allogeneic MHC molecules, or cytokines like IL-2. Because of the serious toxicity of systemically administered IL-2 observed in clinical practice [17] it can be expected that local expression of IL-2 is less harmful to the patient than systemic administration to trigger the immune system. In this regard, adenoviral-mediated expression of IL-2 cytokine gene in several tumor models has been found to induce strong and specific antitumor responses [18] by stimulating immune cells including T and natural killer cells. But adenoviral transfection may raise safety questions in human gene therapy. Therefore, we were interested in evaluating the potential of IL-2 non-viral transfected CIK cells for their ability to stimulate and activate immunologic effector cells.

The use of gene transfected lymphocytes were hampered by a poor efficiency of gene transfer in lymphocytes and a down regulation of cytokine expression [19]. In contrast, nucleofection is a fast and cost-effective method for transfection of large amounts of cells. Here, nucleofection resulted in a significant higher production of IL-2 compared to mock transfected CIK cells (*P *= 0.05). This results matches perfect to a previously reported result by our group [8]. To the best of our knowledge no further report about nucleofection of CIK cells were available. We then showed, that transfection of CIK cells with IL-2 enhances cytotoxic activity (Fig. 1) compared to non-transfected CIK cells co-cultured with DC. No significant cytotoxic activity was seen when DC were pulsed with tumor lysate of Dan-G cells were used with IL-2 transfected CIK cells. This may be due to inhibitory factors in the tumor lysate which may contribute to a decrease in lytic activity.

This effect is due to increased amounts of CIK cells during co-cultivation of IL-2 secreting CIK cells with DC. IL-2 secretion by the CIK cells enhances the NK cell antitumor activity [20]. NK cells proliferate in the presence of IL-2 [21]. This led to a higher amount of effector cells resulting in a higher cytotoxic activity. This effect of inducing proliferation of tumoricidal lymphocytes is well known and the most important biologic effect of IL-2 on immune cells. The reproducible observation that virtually all malignant cells can be lysed by IL-2 stimulated lymphocytes in a manner directly related to the intensity of IL-2 administration encouraged the pursuit of aggressive, intensive clinical trials, especially in renal cell carcinoma and melanoma. Several authors have shown the efficacy of transfecting primary tumor cells and tumor cell lines with plasmid DNA/lipid complexes [22]. Local production of high concentrations of IL-2 and IFN-alpha at the tumor site was more effective in preventing tumor growth than systemic administration in patients with metastatic renal cell carcinoma [22]. There are several reports introducing IL-2 producing genes into pancreatic cancer, but there are no reports about IL-2 secreting lymphocytes functioning as immune enhancer cells. Therefore, our report is the first describing CIK cells to have enhanced in vitro cytolytic activity via non-viral interleukin-2 gene transfer against pancreatic cancer cell lines. Direct delivery of plasmid IL-2 gene to the established tumors in mice showed an increase in both early and long term survival [3]. Preclinical efficacy studies in a renal cell carcinoma, murine model also showed that direct intra-tumoral administration of an IL-2 plasmid DNA/DMRIE/DOPE complex resulted in the generation of tumor specific lymphocytes and complete tumor regression [23]. It is reasonable too, that these effector cells given in a pancreatic carcinoma model should enhance cytotoxic activity. These investigations are ongoing.
